# AI-assisted clinico–quantitative imaging nomogram for preoperative malignancy risk in solid and part-solid pulmonary nodules ≤ 3 cm: development and internal validation

**DOI:** 10.3389/fonc.2026.1754582

**Published:** 2026-04-13

**Authors:** Yingding Ruan, Chuan Long, Wenjun Cao, Jianwei Han, Hongsheng Xue, Aiming Yang, Peng Sun, Ting Zhang

**Affiliations:** 1 Department of Thoracic Surgery, the First People’s Hospital of Jiande, Jiande, China; 2 Department of Thoracic Surgery, Affiliated Zhongshan Hospital of Dalian University, Dalian, China; 3 Department of Radiology, the First People’s Hospital of Jiande, Jiande, China; 4 Radiotherapy Department, Second Affiliated Hospital, Zhejiang University School of Medicine, Hangzhou, China

**Keywords:** artificial intelligence, nomogram, pulmonary nodule, quantitative imaging, risk prediction

## Abstract

**Purpose:**

To develop and internally validate an artificial intelligence (AI)–assisted clinico–quantitative imaging prediction model that combines automatically extracted quantitative imaging features with clinical data to preoperatively assess individualized malignancy risk in solid and part-solid pulmonary nodules (PNs) measuring ≤ 3 cm.

**Methods:**

This retrospective study analyzed data from 951 consecutive patients who underwent surgical resection for PNs ≤ 3 cm (210 benign, 741 malignant). Quantitative CT features, including nodule size, minimum and maximum computed tomography attenuation, consolidation-to-tumor ratio, and nodule type, were automatically measured using InferRead CT Lung AI software (Infervision, Beijing, China; version 4.0). These AI-assisted quantitative measurements were evaluated together with clinical variables and inflammatory markers as candidate predictors. The final prediction model was a multivariable logistic regression model, with predictor selection performed in the full cohort before internal bootstrap validation. Model performance was assessed using 1,000 bootstrap resamples, with discrimination quantified by the area under the receiver operating characteristic curve (AUC), calibration assessed by calibration plots, slope, intercept, and mean absolute error, and clinical utility evaluated using decision curve analysis.

**Results:**

The final AI-assisted model demonstrated strong discrimination, with an AUC of 0.836 (95% confidence interval [CI], 0.804–0.869), and excellent calibration, with a mean absolute error of 0.015. Decision curve analysis indicated a meaningful net clinical benefit across threshold probabilities of 0.10–0.45. Risk stratification based on quartiles of predicted probability categorized patients into lower-, intermediate-, and higher-risk strata, with observed malignancy rates of 43.3%, 86.7%, and 95.0%, respectively, in this surgically managed cohort. The interactive calculator is publicly accessible at https://ruanyingding.shinyapps.io/myshinyapp/. Because the study cohort was restricted to surgically resected nodules, these risk estimates should be interpreted within a malignancy-enriched preoperative surgical setting.

**Conclusion:**

An AI-assisted clinico–quantitative imaging nomogram was developed and internally validated to support individualized preoperative malignancy risk assessment for indeterminate PNs ≤ 3 cm. Because the model was derived in a surgically selected, malignancy-enriched cohort, it is best interpreted as a tool for preoperative surgical decision support rather than for screening or incidental pulmonary nodule populations. External validation and, if necessary, recalibration in independent unselected cohorts are required before broader implementation.

## Introduction

The proven effectiveness of low-dose computed tomography (LDCT) in lung cancer screening, together with advances in artificial intelligence (AI), has substantially increased the detection of indeterminate pulmonary nodules (PNs) measuring ≤3 cm. This cutoff follows the conventional definition of a PN; lesions > 3 cm are typically considered lung masses, which have distinct diagnostic and management pathways and a higher pretest probability of malignancy. The increase in detection presents a clinical challenge in distinguishing benign from malignant lesions prior to surgery (1–3). Although most nodules are benign, with estimates suggesting that 75–80% of detected nodules are non-malignant, many patients still undergo invasive procedures for definitive diagnosis. This highlights the need for more accurate, non-invasive risk assessment tools to reduce unnecessary interventions and improve healthcare efficiency (4–6). Recent studies have shown that a substantial proportion of benign nodules, up to 15–25%, still undergo invasive diagnostic procedures, further underscoring the importance of refining diagnostic strategies to minimize overdiagnosis and avoid unnecessary treatment (7–9). However, surgical candidates represent a malignancy-enriched, higher-pretest-probability subset due to clinical and imaging-based preselection; therefore, models developed in this setting should be interpreted within a preoperative decision-making context.

Although the Fleischner and Lung-RADS guidelines provide a basic framework based on nodule size and density (10, 11), their ability to discriminate malignancy in indeterminate nodules, particularly solid and part-solid lesions measuring 1–3 cm, may be limited, leading to diagnostic uncertainty and variability in clinical practice (12). Consequently, there is growing interest in multivariable models that integrate diverse clinical and radiological data to provide individualized risk assessment (13–15).

AI in radiology offers a promising approach. AI-based software can automatically and reproducibly quantify important imaging features, including nodule size, attenuation (Hounsfield units), and consolidation-to-tumor ratio (CTR), from CT scans, providing an objective and high-throughput alternative to subjective visual assessment (16, 17). At the same time, systemic host factors are increasingly recognized as relevant to the tumor microenvironment. Systemic inflammatory markers derived from routine blood tests reflect host immune status and have been shown to independently predict outcomes in several cancers, including lung cancer (18–21). In addition, diabetes mellitus and elevated glycated hemoglobin (HbA1c) are associated with cancer risk and progression through mechanisms such as chronic inflammation, hyperinsulinemia, and oxidative stress. These factors may hold potential for cancer risk prediction but remain largely underexplored in the context of PNs (22).

Despite these advances, a significant gap remains. Existing models often operate independently without integrating AI-derived quantitative imaging features, systemic inflammatory markers, and metabolic parameters into a unified framework (14, 23, 24). Integrating these complementary data sources may enhance predictive accuracy compared with traditional approaches and address a critical limitation in current predictive modeling strategies.

This study aimed to develop and internally validate an AI-assisted clinico–quantitative imaging nomogram for preoperative malignancy risk estimation in patients with solid and part-solid PNs measuring ≤ 3 cm who were selected for surgical management. A comprehensive set of predictors, including automatically extracted quantitative CT features, clinical characteristics, systemic inflammatory markers, and diabetic status, was evaluated. We specifically sought to develop an interpretable multivariable model for use in the preoperative surgical decision-making context, rather than for screening or incidental pulmonary nodule populations.

## Patients and methods

### Study population

We conducted a single-center retrospective cohort study at the First People’s Hospital of Jiande, enrolling consecutive patients who underwent surgical resection for PNs between January 2012 and July 2025. In routine practice at our thoracic surgery center, surgery follows multidisciplinary assessment using guideline-consistent indications, thereby enriching malignancy prevalence compared with unselected nodules. Accordingly, the present cohort should be viewed as a preoperative surgical population with a substantially higher pretest probability of malignancy than screening-detected or incidentally detected nodule cohorts. All participants underwent preoperative thin-section chest CT with a slice thickness of 1.25–1.5 mm. All scans were non-contrast and used for quantitative imaging analysis. The study protocol was approved by the institutional Ethics Committee (Approval Number: 20251028-KY-002-01), and the requirement for informed consent was waived.

For this analysis, eligibility was restricted to solid nodules (SNs) and part-SNs (PSNs); pure ground-glass nodules (pGGNs) were excluded. The inclusion criteria were: (1) age 18–80 years; (2) histopathologic confirmation after surgical resection; (3) thin-section CT performed within 30 days before surgery; (4) complete clinical records and preoperative laboratory data, including routine blood counts; and (5) a solid or part-solid PN with a maximum diameter ≤ 3 cm on thin-section CT. The exclusion criteria were: (1) any preoperative anticancer treatment (e.g., chemotherapy, radiotherapy, or ablation); (2) active systemic infection or autoimmune disease at the time of evaluation; (3) pregnancy or lactation; (4) reoperation within 30 days; and (5) non-primary lung cancer, including pulmonary metastases or direct invasion from extrapulmonary primaries.

This study is part of a broader research program on PNs. It represents a pre-specified analysis focused on SNs and PSNs and was conducted independently from our all-nodule analysis, with separate ethics approval, validation procedures, and reported endpoints.

### Data collection

Clinical and demographic data, including age, sex, body mass index, smoking history, and comorbidities, were extracted from electronic medical records. Preoperative peripheral blood counts were used to calculate systemic inflammatory indices: neutrophil-to-lymphocyte ratio (NLR), monocyte-to-lymphocyte ratio (MLR), platelet-to-lymphocyte ratio (PLR), and systemic immune-inflammation index (SII), defined as (platelet count × neutrophil count)/lymphocyte count. The time from initial radiologic detection to surgery was calculated and dichotomized as < 2 years versus ≥ 2 years based on clinical management guidelines (10, 11), reflecting the reduced likelihood of malignancy in nodules that remain stable over time (25). Nodule change during follow-up was coded as yes/no and defined as any increase in nodule size or solid component on interval thin-section CT prior to surgery.

### CT Imaging acquisition and evaluation

CT scans were performed with patients in the supine position during an inspiratory breath-hold using the Revolution Apex Expert CT scanner (GE HealthCare, Waukesha, WI, USA). A standardized non-contrast thin-section chest protocol was applied (tube voltage 100–120 kVp; automatic tube current modulation; gantry rotation time 0.35–0.50 s; pitch 0.98–1.38; detector collimation 0.625 mm; matrix 512 × 512; reconstruction with lung and mediastinal kernels at 1.25–1.5-mm slice thickness and 1.0–1.25-mm increment). Preoperative DICOM images were analyzed using a dual approach. All quantitative measurements were derived from non-contrast thin-section CT images. Quantitative imaging data were automatically generated by AI software and recorded by the study team; the reference standard for malignancy was histopathology.

PNs were categorized into three types based on established radiologic criteria (7, 21). As this study focused on malignancy prediction in SNs and PSNs, only SNs and PSNs were included, and pGGNs were excluded.

First, key AI-derived quantitative imaging features—including nodule size, nodule volume (mm³), axial area (mm²), minimum and maximum CT attenuation (HU), CTR, and nodule type (solid vs part-solid)—were automatically measured using InferRead CT Lung AI software (Infervision, Beijing, China; version 4.0). In this study, the term “quantitative imaging features” refers to automatically extracted, interpretable CT-derived metrics rather than high-dimensional texture-based radiomics features. The software performed automated nodule segmentation and quantitative measurement. All automated segmentations and measurements were independently reviewed by a thoracic surgeon and a thoracic radiologist, and manual correction was performed by consensus when necessary. This review was conducted for quality control of AI-derived outputs and not for independent malignancy classification. Nodule size was defined as the maximum axial diameter; nodule volume and axial area were computed from the segmented lesion; and minimum/maximum CT attenuation were defined as voxel-wise HU extrema within the segmentation mask. Thus, in the present study, AI was used to support automated segmentation and extraction of interpretable quantitative imaging variables, whereas malignancy prediction was performed using a conventional multivariable logistic regression framework.

Second, all CT scans were independently evaluated by a thoracic surgeon and a thoracic radiologist blinded to pathological results. Disagreements were resolved by consensus. Qualitative imaging features were defined according to the Fleischner Society glossary (26, 27). This qualitative review was performed to document prespecified morphologic features and support imaging quality control; it did not generate a standalone benign/malignant classification for comparison with the AI model.

CTR was dichotomized as ≤ 0.5 versus > 0.5, reflecting its association with pathological invasiveness (28, 29). Suspicious morphologic features—including lobulation, spiculation, pleural retraction, and cavitation—were evaluated according to standard radiologic definitions (11, 30). “Suspicious radiologic features” were defined as the presence of any of these findings and were coded as present versus absent.

### Statistical analysis

Statistical analyses were conducted using R software (version 4.1.2) and SPSS (version 22.0). Continuous variables are presented as mean ± standard deviation or median (interquartile range), and categorical variables are presented as frequencies (percentages). Normality was assessed using the Shapiro–Wilk test. Comparisons between benign and malignant nodules were performed using Student’s t-test, Mann–Whitney U test, χ² test, or Fisher’s exact test, as appropriate.

Multicollinearity was evaluated using the variance inflation factor (VIF). A VIF > 10 indicated severe multicollinearity (31). Predictors included in the final model did not exhibit severe multicollinearity (**Supplementary Table 1**).

Candidate variables were initially screened using univariable analyses (P < 0.05), after which final predictors were selected based on statistical significance together with clinical and radiologic plausibility to preserve interpretability. It is important to clarify that while AI software was employed for the automated and objective extraction of quantitative imaging features, the predictive model itself is a conventional multivariable logistic regression, not an end-to-end deep learning classifier. Categorical variables were modeled in full to maintain clinical relevance (32). The final model was constructed by simultaneously entering all selected predictors. For interpretability, selected continuous variables were scaled to clinically meaningful units before inclusion: minimum and maximum CT attenuation (per 100 HU), nodule volume (per 1000 mm³), and nodule area (per 500 mm²).

Model performance was assessed in terms of discrimination, calibration, and clinical utility. Discrimination was quantified using AUC; calibration was evaluated using calibration plots and mean absolute error. The full model was benchmarked against prespecified clinical-only, imaging baseline, and guideline-like imaging models in the same cohort using AUC (95% CI) and DeLong’s test. Internal validation was performed using bootstrap resampling (1,000 iterations) to estimate optimism (33). Variable selection was conducted once in the full cohort prior to bootstrapping, rather than being repeated within each bootstrap sample. We acknowledge that this approach may lead to an underestimation of model optimism, which is a limitation of our validation strategy. No external validation dataset was available. Clinical utility was evaluated using decision curve analysis (DCA) across clinically relevant high-risk thresholds to determine the net benefit of applying the model in clinical decision-making (34, 35).

A nomogram was constructed from the final multivariable logistic regression model. The full prediction formula is provided in **Supplementary Appendix S2**. All statistical tests were two-sided, and *P* < 0.05 was considered statistically significant.

## Results

### Study population and baseline characteristics

The final analysis included 951 patients who underwent surgical resection of solid or part-solid PNs measuring ≤ 3 cm. The cohort comprised 741 (77.9%) malignant and 210 (22.1%) benign lesions. This malignancy prevalence reflects the preselected surgical cohort and should not be interpreted as the malignancy prevalence of incidentally detected PNs. **Table 1** presents the baseline clinicopathological and radiological characteristics of the cohort, stratified by pathological outcome.

**Table 1:** Comparison of clinicopathological characteristics between benign and malignant solid and part-solid pulmonary nodules [n(%), Mean ± SD, M(P25, P75)].

Variables	Total (n = 951)	Benign (n = 210)	Malignancy (n = 741)	*P*	SMD
Sex, n(%)				< 0.001	0.278
Male	401 (42.2)	111 (52.9)	290 (39.1)		
Female	550 (57.8)	99 (47.1)	451 (60.9)		
Smoking History (yes), n(%)	257 (27.0)	59 (28.1)	198 (26.7)	0.692	0.031
Diabetes Mellitus (yes), n(%)	110 (11.6)	23 (11.0)	87 (11.7)	0.752	0.025
HbA1c, n(%)				0.231	0.092
<6.5	804 (84.5)	172 (81.9)	632 (85.3)		
≥ 6.5	147 (15.5)	38 (18.1)	109 (14.7)		
Age, years (Mean ± SD)	61.5 ± 10.4	59.8 ± 9.8	62.0 ± 10.5	0.006	0.221
BMI, kg/m^2^ (Mean ± SD)	23.3 ± 3.6	23.6 ± 3.8	23.2 ± 3.6	0.176	0.104
Symptoms at Detection, n(%)				0.001	0.243
Asymptomatic	710 (74.7)	139 (66.2)	571 (77.1)		
Symptomatic	241 (25.3)	71 (33.8)	170 (22.9)		
Nodule Change During Follow-up, n(%)				0.371	0.071
NO	777 (81.7)	176 (83.8)	601 (81.1)		
YES	174 (18.3)	34 (16.2)	140 (18.9)		
Time from Detection to Surgery, n(%)				< 0.001	0.320
< 2 years	803 (84.4)	157 (74.8)	646 (87.2)		
≥ 2 years	148 (15.6)	53 (25.2)	95 (12.8)		
Resection Side, n(%)				0.520	0.051
Right	575 (60.5)	131 (62.4)	444 (59.9)		
Left	376 (39.5)	79 (37.6)	297 (40.1)		
Nodule Radiographic Appearance, n(%)				< 0.001	0.700
Part-solid nodule	501 (52.7)	57 (27.1)	444 (59.9)		
Solid nodule	450 (47.3)	153 (72.9)	297 (40.1)		
Consolidation-to-Tumor, n(%)				< 0.001	0.566
0.01–0.50	358 (37.6)	38 (18.1)	320 (43.2)		
> 0.50	593 (62.4)	172 (81.9)	421 (56.8)		
Suspicious Radiologic Features, n(%)				< 0.001	0.826
Absent	284 (29.9)	124 (59.0)	160 (21.6)		
Present	667 (70.1)	86 (41.0)	581 (78.4)		
Nodule Size, n (%)				< 0.001	0.418
≤ 1 cm	162 (17.0)	51 (24.3)	111 (15.0)		
> 1 cm, ≤ 2 cm	467 (49.1)	117 (55.7)	350 (47.2)		
> 2 cm, ≤ 3 cm	322 (33.9)	42 (20.0)	280 (37.8)		
Max CT Attenuation (HU), M(P25, P75)	328 (157, 437)	343 (223, 434)	319 (142, 437)	0.019	0.255
Min CT Attenuation(HU), M(P25, P75)	−586 (−706, −389)	−452 (−623, −300)	−607 (−713, −451)	< 0.001	0.487
Nodule Volume (mm^3^), M(P25, P75)	687 (272, 1955)	598 (266, 1494)	754 (274, 2142)	0.019	0.235
Nodule Area (mm^2^), M(P25, P75)	597 (283, 1211)	494 (284, 980)	624 (283, 1298)	0.021	0.223
Nodule Mass (g), M(P25, P75)	528 (189, 1541)	524 (213, 1423)	531 (188, 1616)	0.800	0.149
Preoperative SII, M(P25, P75)	437.7 (322.9, 619.9)	480.7 (338.2, 676.6)	432.0 (315.4, 601.7)	0.013	0.187
Preoperative PLR, M(P25, P75)	127.2 (97.3, 162.2)	133.6 (100.8, 168.9)	124.2 (96.5, 160.8)	0.100	0.148
Preoperative NLR, M(P25, P75)	2.5 (1.8, 3.4)	2.6 (1.9, 3.6)	2.4 (1.8, 3.3)	0.026	0.122
Preoperative LMR, M(P25, P75)	4.0 (3.0, 5.3)	4.0 (2.7, 5.0)	4.1 (3.0, 5.3)	0.059	0.135
Preoperative ALB, g/L, Mean ± SD	43.3 ± 4.1	43.1 ± 4.0	43.4 ± 4.2	0.325	0.078

BMI, Body Mass Index; HbA1c, Glycated Hemoglobin; CTR, Consolidation-Tumor Ratio; HU, Hounsfield Units; SII, Systemic Immune-Inflammation Index; PLR, Platelet-to-Lymphocyte Ratio; NLR, Neutrophil-to-Lymphocyte Ratio; LMR, Lymphocyte-to-Monocyte Ratio; ALB, Albumin. M(P25, P75), median (25th percentile, 75th percentile) Note: Data are presented as n (%), mean ± standard deviation, or median (25th percentile, 75th percentile) as appropriate.

Suspicious radiologic features were defined as the presence of any of the following morphologic features on thin-section CT: lobulation, spiculation, pleural retraction, or cavitation (present vs. absent)

Time from detection to surgery was categorized as < 2 years vs. ≥ 2 years.

Nodule change during follow-up was defined as any radiologic progression on interval CT (increase in nodule size and/or increase in the solid component)

Smoking history and diabetes mellitus were evaluated as binary variables (yes/no) for all patients.

Max CT attenuation represents the voxel-wise maximum HU within the AI-segmented nodule mask on non-contrast thin-section CT.

Several key variables differed significantly between benign and malignant nodules. Patients with malignant nodules were more often female (60.9% vs. 47.1%; *P* < 0.001) and asymptomatic at the time of detection (77.1% vs. 66.2%; *P* = 0.001). Malignant nodules were also more likely to have a shorter interval from detection to surgery (< 2 years: 87.2% vs. 74.8%; *P* < 0.001), a mixed radiographic appearance (59.9% vs. 27.1%; *P* < 0.001), a consolidation-to-tumor ratio (CTR) ≤ 0.5 (43.2% vs. 18.1%; *P* < 0.001), and the presence of suspicious radiologic features (78.4% vs. 41.0%; *P* < 0.001). Furthermore, malignant nodules exhibited significantly lower minimum CT attenuation (median −607 HU vs. −452 HU; *P* < 0.001) and a lower SII (median 432.0 vs. 480.7; *P* = 0.013).

### Predictor selection and final prediction model

Univariable and multivariable logistic regression analyses were conducted to identify independent predictors of malignancy (**Table 2**). The final multivariable model was constructed by jointly considering statistical significance and clinical/radiological relevance. An evaluation of multicollinearity among candidate predictors is provided in **Supplementary Table 1**. The complete prediction formula for calculating the linear predictor and individual malignancy probability is provided in **Supplementary Appendix S2**.

**Table 2:** Univariate and multivariable analysis for predictors of malignancy in solid and part-solid pulmonary nodules.

Variables	Univariate logistic regression analyses					Multivariable logistic regression analyses				
	Estimate	S.E.	Z	*P*	Adjusted OR (95% CI)	Estimate	S.E.	Z	*P*	Adjusted OR (95% CI)
Sex										
Male	Ref					Ref				
Female	0.56	0.16	3.53	< 0.001	1.74 (1.28–2.38)	0.6	0.2	3.06	0.002	1.82 (1.24–2.67)
Smoking History	−0.07	0.17	−0.40	0.692	0.93 (0.67–1.32)					
Diabetes Mellitus	0.08	0.25	0.32	0.752	1.08 (0.68–1.80)					
HbA1c										
< 6.5	Ref									
≥ 6.5	−0.25	0.21	−1.20	0.232	0.87 (0.61–1.27)					
Age	0.02	0.01	2.75	0.006	1.02 (1.01–1.04)	0.01	0.01	0.88	0.380	1.01 (0.99–1.03)
BMI	−0.03	0.02	−1.35	0.176	0.97 (0.93–1.01)					
Symptoms at detection										
Asymptomatic	Ref					Ref				
Symptomatic	−0.54	0.17	−3.18	0.002	0.58 (0.42–0.82)	−0.62	0.21	−2.92	0.004	0.54 (0.35–0.82)
Nodule Change During Follow-up										
No	Ref									
Yes	0.19	0.21	0.89	0.372	1.21 (0.81–1.84)					
Time from Detection to Surgery										
<2 years	Ref					Ref				
≥ 2 years	−0.83	0.19	−4.30	< 0.001	0.44 (0.30–0.64)	−0.6	0.24	−2.46	0.014	0.55 (0.34–0.89)
Resection Side										
Right	Ref									
Left	0.1	0.16	0.64	0.52	1.11 (0.81–1.52)					
Nodule radiographic appearance										
Part-solid nodule	Ref					Ref				
Solid nodule	−1.39	0.17	−8.06	< 0.001	0.25 (0.18–0.35)	−0.58	0.29	−2.01	0.045	0.56 (0.31–0.98)
Consolidation-to-tumor										
0.01–0.50	Ref					Ref				
> 0.50	−1.24	0.19	−6.37	< 0.001	0.29 (0.20–0.42)	−1.11	0.32	−3.48	< 0.001	0.33 (0.18–0.61)
Suspicious radiologic features										
Absent	Ref					Ref				
Present	1.66	0.17	9.95	< 0.001	5.24 (3.79–7.27)	2.08	0.22	9.34	< 0.001	7.98 (5.20–12.45)
Nodule Size										
≤ 1 cm	Ref					Ref				
> 1 cm, ≤ 2 cm	0.32	0.2	1.59	0.112	1.37 (0.92–2.03)	0.01	0.26	0.03	0.978	1.01 (0.60– 1.67)
> 2 cm,≤ 3 cm	1.12	0.24	4.73	< 0.001	3.06 (1.93–4.89)	1.13	0.36	3.14	0.002	3.10 (1.52– 6.27)
Max CT Attenuation (per 100 HU)	−0.06	0.02	−3.49	< 0.001	0.94 (0.90–0.97)	−0.05	0.02	−2.20	0.028	0.95 (0.91– 0.99)
Min CT Attenuation (per 100 HU)	−0.23	0.04	−6.05	< 0.001	0.80 (0.74–0.86)	−0.21	0.06	−3.41	< 0.001	0.81 (0.72– 0.91)
Nodule Volume (per 1000 mm^3^)	0.14	0.05	2.93	0.003	1.16 (1.06–1.28)	0.03	0.05	0.61	0.544	1.03 (0.93– 1.18)
Nodule Area (per 500 mm^2^)	0.18	0.06	3.16	0.002	1.20 (1.08–1.35)	0.00	0.04	0.02	0.983	1.00 (0.93– 1.19)
Nodule Mass (per 500 mg)	0.04	0.02	1.68	0.094	1.04 (1.00–1.09)					
Preoperative SII (per 100)	−0.06	0.02	−2.47	0.014	0.95 (0.91–0.99)	−0.04	0.03	−1.30	0.195	0.96 (0.91– 1.02)
Preoperative PLR (per 50)	−0.11	0.06	−1.95	0.052	0.90 (0.80–1.00)					
Preoperative NLR	−0.06	0.04	−1.58	0.114	0.94 (0.87–1.02)					
Preoperative LMR	0.08	0.04	1.73	0.083	1.08 (0.99–1.18)					
Preoperative ALB	0.02	0.02	0.98	0.325	1.02 (0.98–1.06)					

Multivariable model corresponds to the final model used for nomogram; blank cells indicate variables not entered into the multivariable model.

The model identified female sex (adjusted odds ratio [aOR] = 1.81, *P* = 0.002) and suspicious radiologic features (aOR = 8.43, *P* < 0.001) as strong positive predictors of malignancy. Nodule size > 2–3 cm (vs. ≤ 1 cm) was also a significant positive predictor (aOR = 3.31, *P* = 0.001). Factors associated with a lower likelihood of malignancy included the presence of symptoms at detection (aOR = 0.53, *P* = 0.003), time to surgery ≥ 2 years (aOR = 0.55, *P* = 0.014), SN appearance (vs. mixed; aOR = 0.54, *P* = 0.035), CTR > 0.5 (aOR = 0.33, *P* < 0.001), and higher minimum (aOR = 0.82 per 100 HU, *P* = 0.001) and maximum CT attenuation values (aOR = 0.95 per 100 HU, *P* = 0.028). The full set of coefficients and 95% confidence intervals is provided in **Table 2**. The complete prediction formula for computing the linear predictor and individual malignancy probability is provided in **Supplementary Appendix S2**.

Although the SII was significantly associated with malignancy in univariable analysis (**Table 1**), along with other inflammatory markers (NLR, PLR, and MLR), it did not remain independently associated after multivariable adjustment and was therefore excluded from the final parsimonious model (**Table 2**).

### Model performance and validation

The overall performance of the final model is presented in **Table 3**. The model demonstrated excellent goodness of fit (likelihood ratio chi-square = 277.02, *P* < 0.0001) and accounted for a substantial proportion of variance (R² = 0.388). A Brier score of 0.117 indicates good overall predictive accuracy.

**Table 3:** Model statistical summary and discrimination metrics.

Observation indicators	Value	Likelihood ratio test	Value	Discrimination indexes	Value	Rank discrim. indexes	Value
Total Observations	951	LR chi2 (likelihood ratio chi-square)	277.02	R2	0.388	C	0.836
Benign Lesion	210	d.f. (degrees of freedom)	10	R2(9,994)	0.245	Dxy	0.672
Malignancy	741	Pr(> chi2) (chi-square test *P* value)	< 0.0001	R2(9,459.2)	0.420	gamma	0.672
max |deriv|	1e−07	-	-	Brier (Brier Score)	0.117	tau-a	0.232

**Figure 1:**
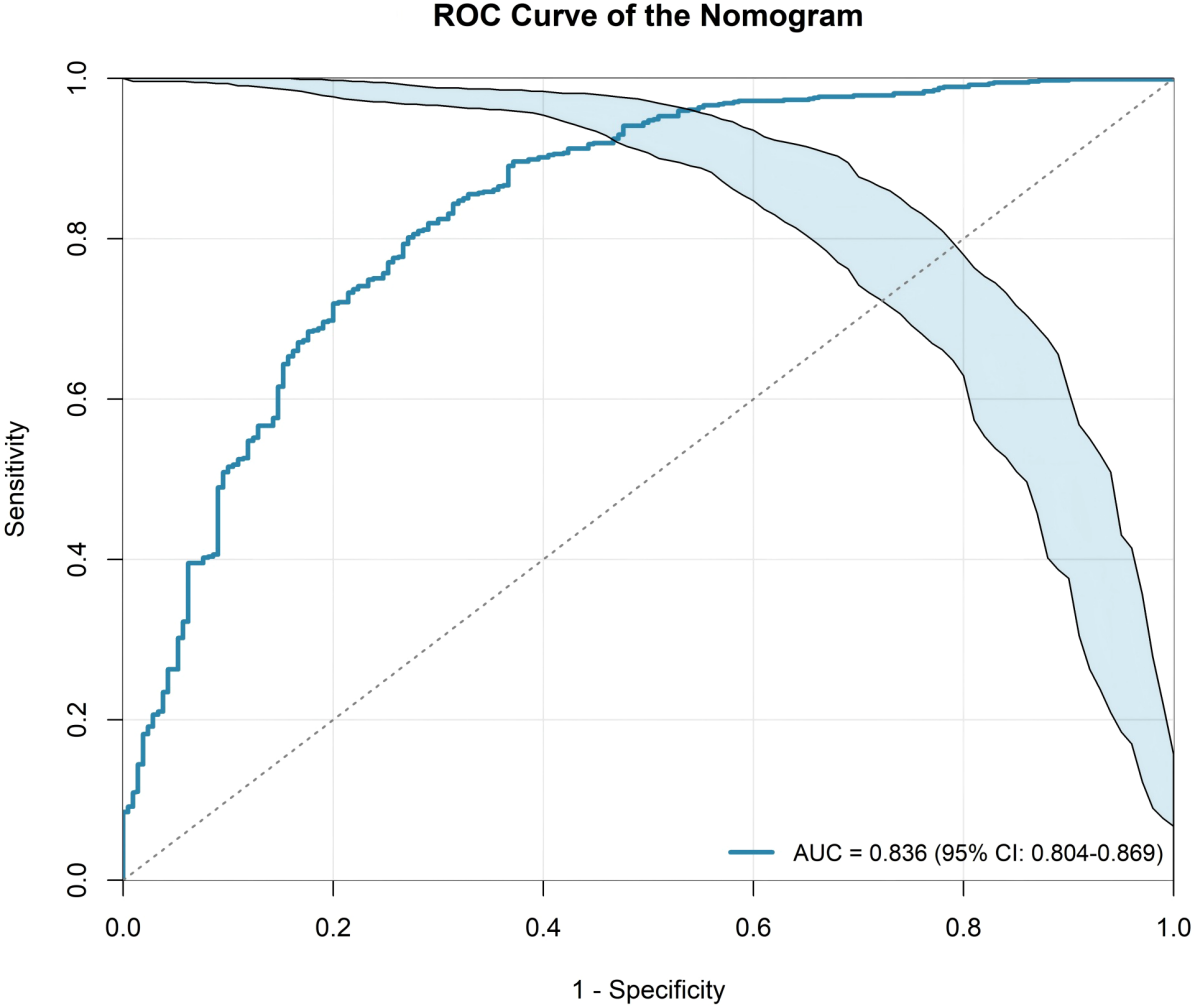
Receiver operating characteristic (ROC) curve of the AI-assisted clinico–quantitative imaging nomogram for preoperative discrimination of malignancy in solid and part-solid pulmonary nodules ≤ 3 cm. The model achieved an area under the curve (AUC) of 0.836 (95% confidence interval [CI], 0.804–0.869). The shaded band denotes the 95% CI for sensitivity across the range of 1–specificity; the diagonal dashed line indicates no discrimination.

The model demonstrated strong discriminatory ability, with an area under the curve (AUC) of 0.836 (95% CI: 0.804–0.869) for the entire cohort (**Figure 1**).

**Figure 2:**
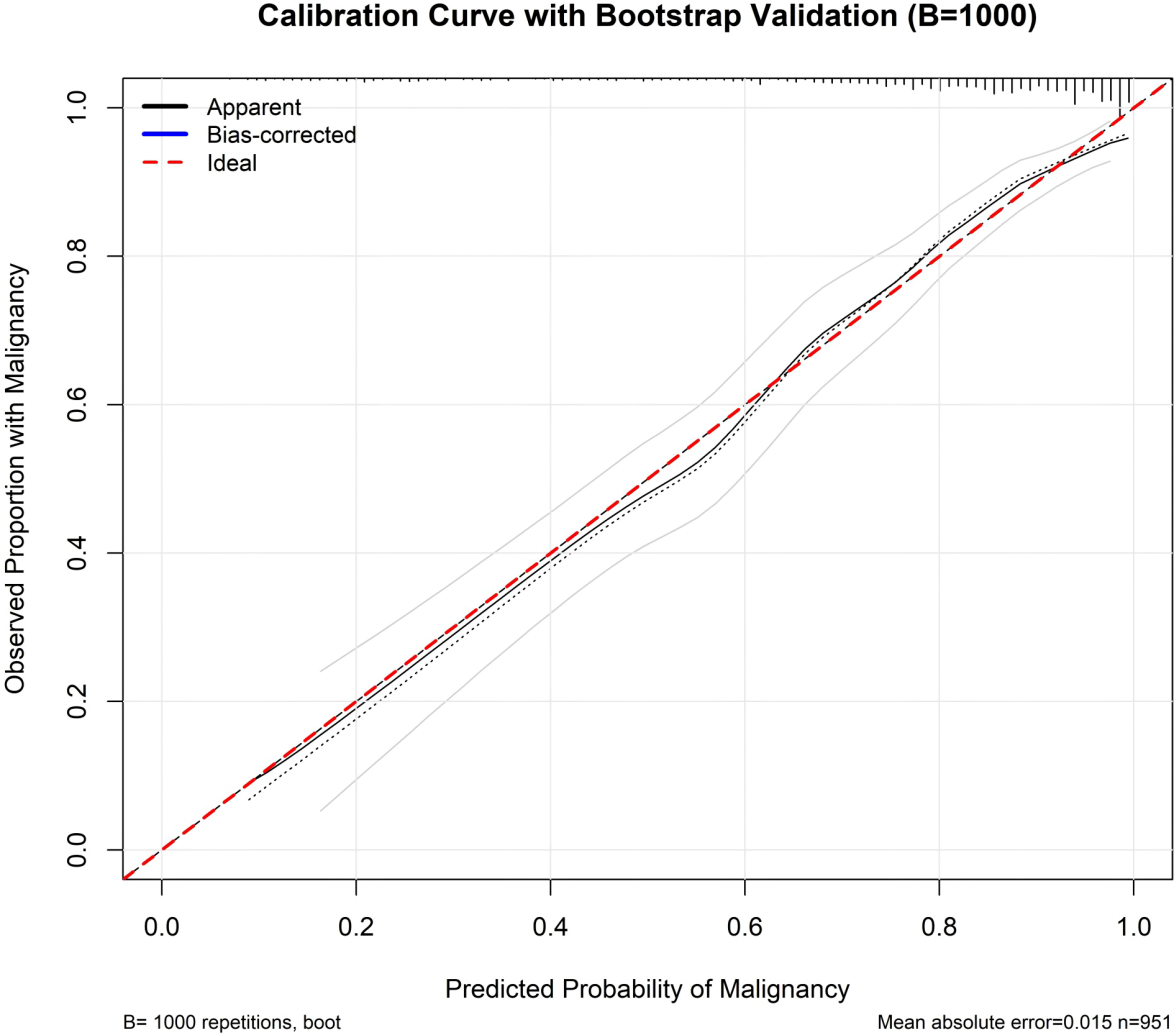
Calibration of the AI-assisted clinico–quantitative imaging nomogram. The plot compares the predicted probabilities of malignancy with observed proportions. The black solid curve (“Apparent”) shows the model fitted to the full dataset; the blue solid curve (“Bias-corrected”) is optimism-adjusted using 1,000 bootstrap resamples of the final model. The red dashed line (“Ideal”) indicates perfect calibration. Rug marks at the top display the distribution of the predicted risks. The mean absolute calibration error was 0.015 (n = 951).

The model also exhibited excellent calibration. The calibration plot demonstrated close agreement between predicted malignancy probabilities and observed outcomes across the full range of risk (**Figure 2**). Based on 1000 bootstrap repetitions, the bias-corrected curve closely followed the ideal reference line, indicating minimal overfitting. Quantitatively, the model showed a low mean absolute error of 0.015, with a bootstrap-corrected calibration slope of 0.98 and an intercept of 0.02, indicating excellent agreement between predicted probabilities and observed outcomes with minimal systematic over- or under-estimation.

### Internal benchmarking against baseline models

In internal benchmarking analyses (**Supplementary Table 2**), the full clinico-quantitative imaging model achieved the highest discrimination (AUC = 0.838), outperforming the clinical-only model (AUC = 0.666; *P* < 0.001) and the guideline-like imaging model (AUC = 0.733; *P* < 0.001). The imaging baseline model showed discrimination close to that of the full model (AUC = 0.822), but the full model remained statistically superior (*P* = 0.017).

### Clinical utility and risk stratification

**Figure 3:**
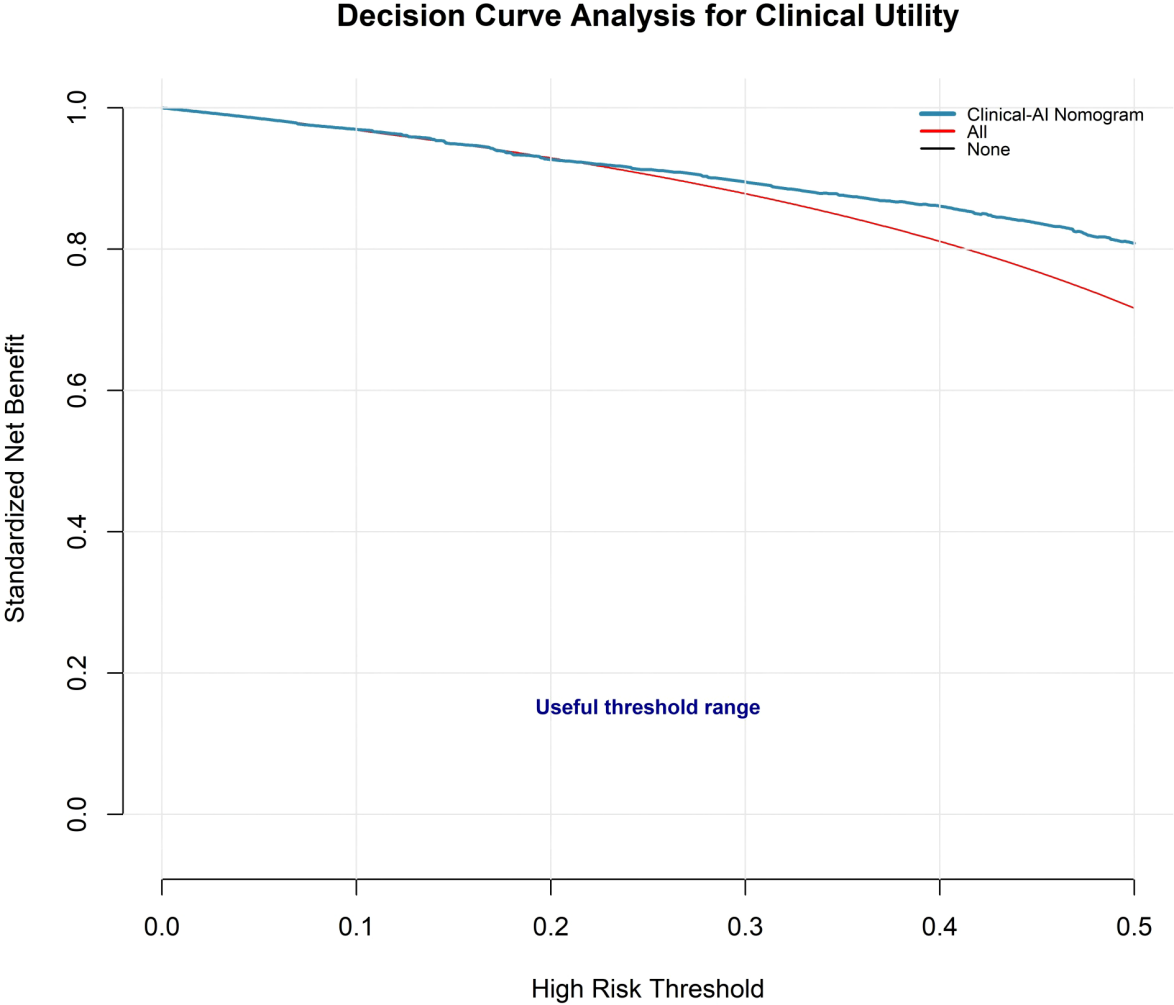
Decision curve analysis of the AI-assisted clinico–quantitative imaging nomogram. The model curve shows the standardized net benefit across threshold probabilities ranging from 0 to 0.50. Compared with the treat-all and treat-none strategies, the nomogram provides a higher net benefit over most clinically relevant thresholds (0.10–0.45), supporting its potential clinical utility. The curves were estimated without confidence intervals.

**Figure 4:**
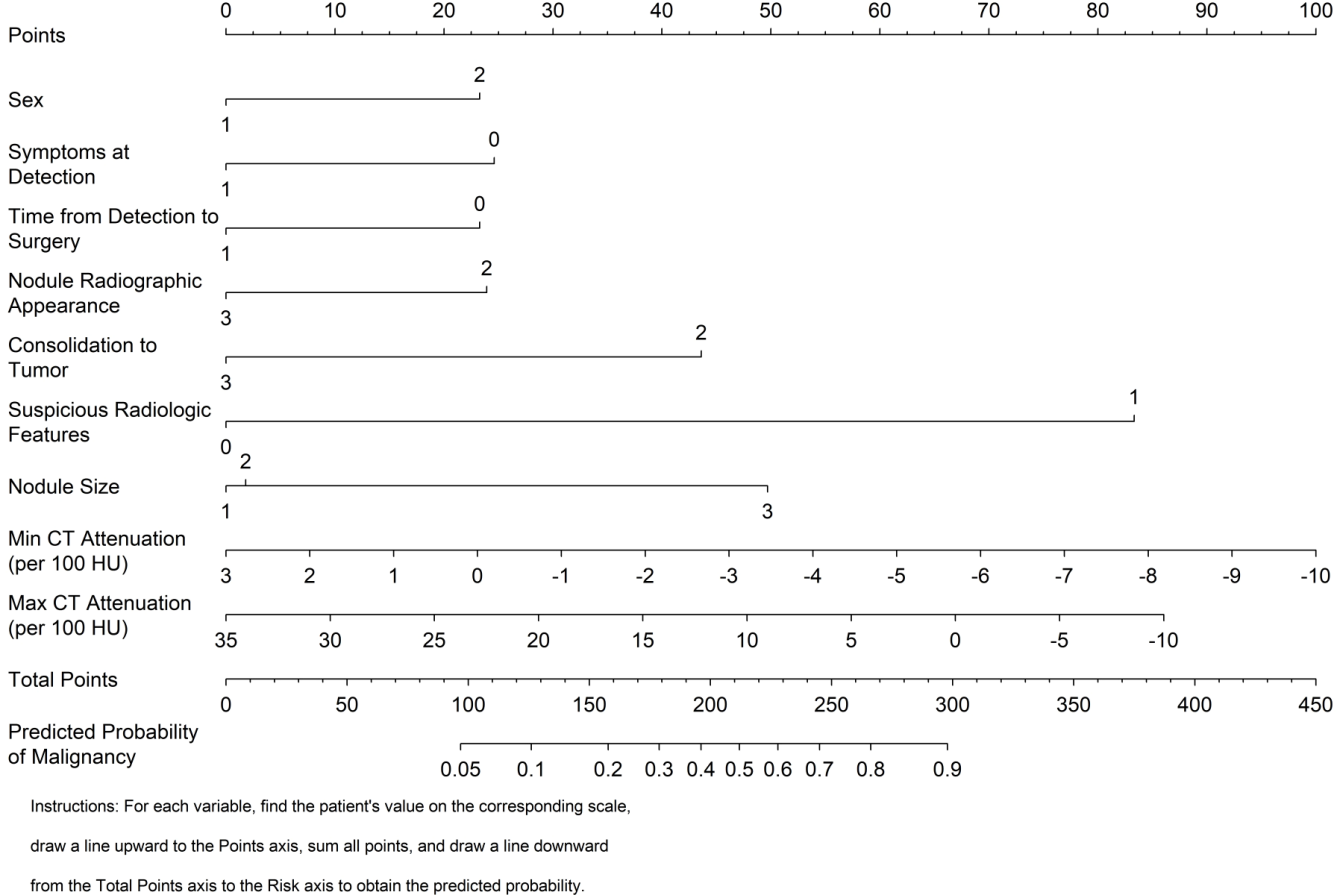
Observed malignancy rates by model-defined predicted-risk strata. The lower, intermediate, and higher predicted-risk strata, defined by quartiles of predicted malignancy probability, showed malignancy rates of 43.3%, 86.7%, and 95.0%, respectively, demonstrating a monotonic increase in observed malignancy and supporting the nomogram’s risk stratification in this surgically managed cohort.

DCA indicated that the model provided greater clinical benefit than strategies of treating all patients or treating none across a broad range of clinically relevant threshold probabilities (approximately 0.10–0.45) (**Figure 3**). The model successfully stratified patients into three distinct model-defined predicted-risk strata with clearly different malignancy rates (**Table 4**, **Figure 4**). Risk strata were defined by quartiles of predicted malignancy probability for descriptive purposes rather than predefined clinical thresholds. The observed malignancy rate was 43.3% in the lower predicted-risk stratum (lowest 25% of predicted probabilities, n = 238), 86.7% in the intermediate predicted-risk stratum (middle 50%, n = 475), and 95.0% in the higher predicted-risk stratum (highest 25%, n = 238). Because this cohort comprised patients selected for surgical evaluation, even the lower predicted-risk stratum retained a substantial malignancy prevalence. These findings highlight the practical value of the nomogram in guiding preoperative clinical decision-making.

**Table 4:** Malignancy rates and predicted risk by model-defined risk category.

Risk_stratum	Patients	Malignant_cases	Malignancy_rate	Mean_predicted_probability	Probability_range
Low predicted-risk strata	238	103	43.3	0.445	Lowest 25%
Intermediate predicted-risk strata	475	412	86.7	0.85	Middle 50%
High predicted-risk strata	238	226	95	0.973	Highest 25%

### Subgroup analysis

Subgroup analyses demonstrated that the model maintained strong and consistent discriminatory performance across key patient and nodule characteristics (**Table 5**). The AUC remained high across subgroups defined by nodule size (≤ 2 cm: AUC = 0.846; > 2 cm: AUC = 0.761), nodule type (solid: AUC = 0.826; mixed: AUC = 0.765), CTR (≤ 0.5: AUC = 0.711; > 0.5: AUC = 0.856), sex, symptom status, and time to surgery, indicating broad applicability of the model.

**Table 5:** Subgroup analysis of malignancy rates and model discrimination (AUC) across clinical and radiologic strata.

Subgroup	Level	N	N_malignant	Malignancy_rate	AUC	AUC_CI
Nodule Size	≤ 2 cm	629	461	73.3	0.846	0.810–0.881
Nodule Size	> 2 cm	322	280	87	0.761	0.681–0.840
CTR	≤ 0.5	358	320	89.4	0.711	0.631–0.792
CTR	> 0.50	593	421	71	0.856	0.822–0.890
Nodule Type	Part-solid nodule	501	444	88.6	0.765	0.694–0.836
Nodule Type	Solid nodule	450	297	66	0.826	0.784–0.867
Gender	Female	550	451	82	0.834	0.786–0.882
Gender	Male	401	290	72.3	0.826	0.778–0.874
Symptoms	Asymptomatic	710	571	80.4	0.812	0.768–0.855
Symptoms	Symptomatic	241	170	70.5	0.879	0.834–0.924
Time to Surgery	≥ 2 years	148	95	64.2	0.809	0.728–0.889
Time to Surgery	< 2 years	803	646	80.4	0.837	0.801–0.874

### Clinical implementation

**Figure 5:**
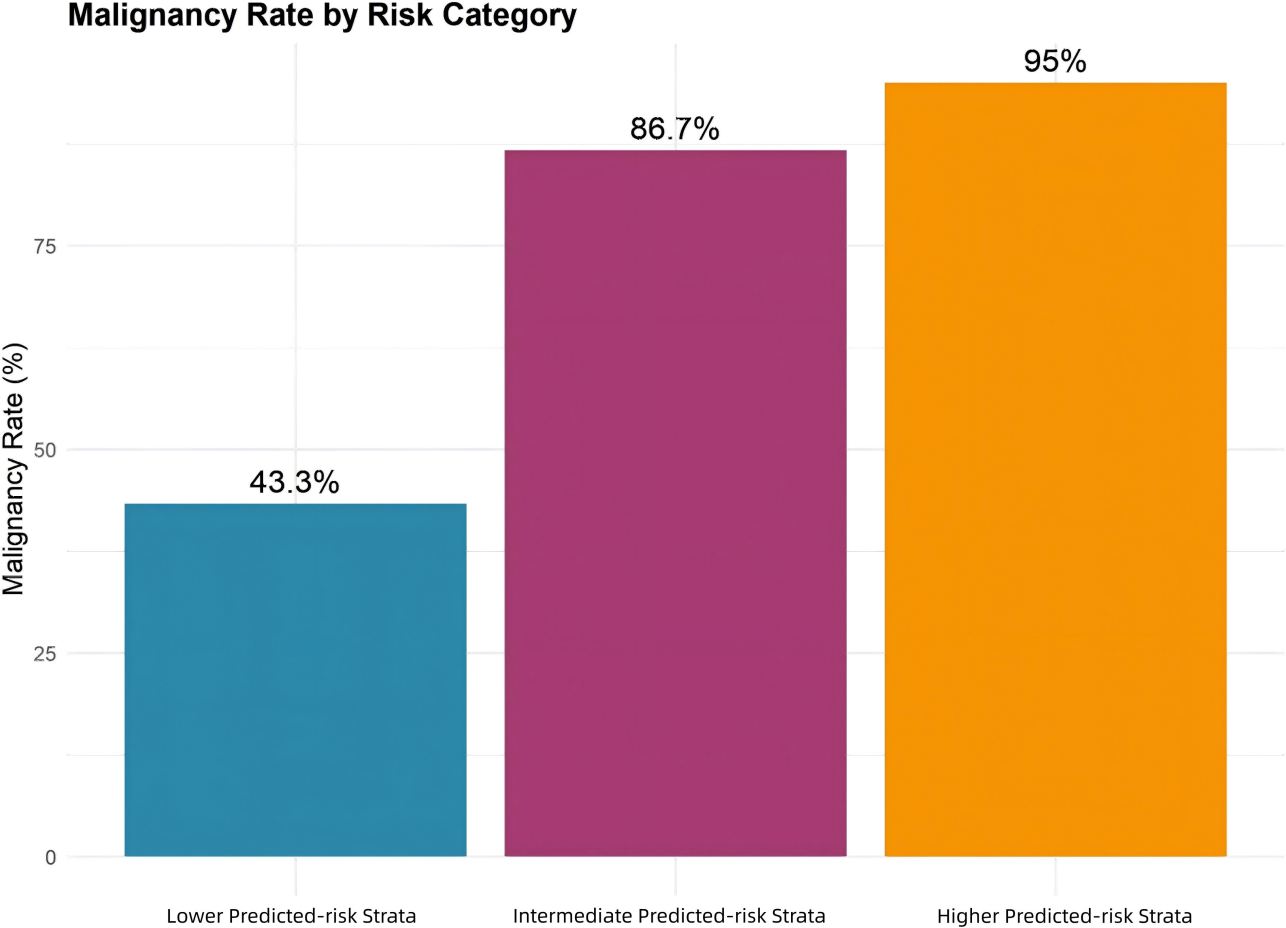
Clinico–quantitative imaging for preoperative prediction of malignancy in pulmonary nodules ≤ 3 cm. Predictors include sex, symptoms at detection, time from detection to surgery, nodule radiographic appearance, consolidation-to-tumor, suspicious radiologic features, nodule size, min CT attenuation (per 100 HU), and max CT attenuation (per 100 HU). To estimate an individual’s risk, locate the patient’s value on each predictor scale, draw a vertical line to the Points axis to assign points, sum the points on the Total Points axis, and project downward to obtain the Predicted Probability of Malignancy. Higher total points indicate a higher risk of malignancy. (model derived from multivariate logistic regression; internal performance is reported in **Figures 1**-**3**).

To support clinical implementation, we developed a user-friendly, point-based nomogram incorporating all significant predictors from the final model (**Figure 5**). This visual tool enables clinicians to rapidly estimate the probability of malignancy for an individual patient. In addition, the model has been made available as an interactive web-based calculator at (https://ruanyingding.shinyapps.io/myshinyapp/).

## Discussion

In this large cohort of surgically resected patients, we developed and internally validated an AI-assisted clinico–quantitative imaging nomogram, which demonstrated strong discrimination (AUC = 0.836), excellent calibration, and greater net clinical benefit than strategies of treating all or no patients across a wide range of clinically relevant thresholds (0.10–0.45). It also effectively stratified patients into risk groups, with a monotonic increase in observed malignancy from 43.3% in the lower predicted-risk stratum to 95.0% in the higher predicted-risk stratum. Although systemic inflammatory indices were assessed, none remained independently associated with malignancy after multivariable adjustment. Consequently, the final model included a streamlined set of clinically and radiologically relevant predictors.

Notably, the observed malignancy rate remained substantial even in the lower predicted-risk stratum (43.3%), reflecting the malignancy-enriched nature of a surgically selected cohort (77.9% overall malignancy prevalence). This highlights the spectrum effect, where prediction tools developed in higher-prevalence clinical settings may yield miscalibrated absolute risks if applied directly to unselected screening or incidental populations (36). In particular, absolute predicted probabilities may differ when the model is applied to populations with substantially different malignancy prevalence. Consequently, our model is most appropriately applied to preoperative risk stratification in patients already deemed candidates for surgical evaluation, rather than for initial triage in screening programs. The lower predicted-risk stratum in our cohort should not be interpreted as a “rule-out” category to defer diagnostic workup. Broader clinical application will require external validation and recalibration in lower-pretest-probability cohorts, with careful attention to calibration and decision thresholds (37, 38).

To contextualize our findings, current PN management frameworks such as the Fleischner Society recommendations and Lung-RADS remain indispensable (10, 11). However, these frameworks rely largely on visual assessment of size and density and may be limited in diagnostically challenging indeterminate nodules (12). AI-based analysis of CT volumes has been shown to improve malignancy discrimination compared with visual assessment alone (39). Building on this rationale, our study translates automated AI-derived quantitative imaging measurements into a transparent, multifactorial prediction model that integrates key imaging metrics with essential clinical variables. In this framework, AI was used to improve the objectivity and reproducibility of quantitative imaging measurements, while prediction remained based on a transparent statistical modeling framework. We also evaluated systemic inflammatory and metabolic host factors as candidate predictors. In our cohort, these host factors showed associations in univariable analyses but did not provide independent predictive value in the final multivariable model.

The final prediction model identified several strong and clinically interpretable independent predictors of malignancy, emphasizing the benefit of integrating multidimensional data. In particular, AI-derived imaging features, including the CTR and minimum and maximum CT attenuation, were significant contributors. The pronounced inverse relationship between higher minimum CT attenuation and malignancy (aOR = 0.82 per 100 HU, *P* = 0.001) is consistent with the established understanding that benign lesions, such as granulomas or intrapulmonary lymph nodes, often exhibit higher density due to fibrosis or calcification, whereas malignant nodules tend to have lower attenuation because of ground-glass components or necrotic regions (40, 41). The automated extraction of these imaging features highlights the important role of AI in improving the objectivity, reproducibility, and quantitative rigor of radiographic assessment, surpassing the limitations of subjective visual interpretation (16, 17). Emerging evidence further demonstrates that combining AI-derived texture-based radiomics features or deep-learning imaging features with other data sources, such as circulating tumor DNA (ctDNA) fragmentomics, can create multimodal models with enhanced diagnostic accuracy and clinical utility for indeterminate PNs, as demonstrated in a recent large multicenter study (42). In future iterations, our nomogram could potentially be adapted to incorporate ctDNA or other biomarkers, further improving its predictive power and clinical applicability.

In this study, several non-imaging predictors also provided valuable insights. Female sex was associated with a higher risk of malignancy (aOR = 1.81, *P* = 0.002). Conversely, symptomatic presentation at detection was associated with a lower risk of malignancy (aOR = 0.53, P = 0.003). This counterintuitive finding may reflect the specific clinical context of our surgical cohort, where symptomatic patients might undergo more aggressive workup leading to the resection of benign inflammatory or infectious lesions that mimic malignancy, whereas asymptomatic nodules detected incidentally and proceeding to surgery are often those with highly suspicious imaging features despite lacking symptoms. In addition, a longer interval from nodule detection to surgery (≥ 2 years) was independently associated with a lower probability of malignancy (aOR = 0.55, *P* = 0.014), consistent with the established concept that long-term nodule stability supports benignity (25). However, it is also important to recognize that the time interval variable may partly reflect clinical decision-making processes and physician confidence in nodule stability, rather than solely representing a biological characteristic of the nodule itself. Given differences across clinical settings and case mix, external validation and recalibration in unselected cohorts are warranted.

In addition to local radiological features, we assessed systemic inflammatory markers, including the SII. At baseline, malignant nodules exhibited lower SII values (median 432.0 vs. 480.7; *P* = 0.013), and SII was associated with malignancy in univariable analysis. This finding also suggests that further studies should evaluate model transportability across different healthcare systems and patient populations. Accordingly, future iterations of our nomogram may incorporate tumor-proximal immune or molecular biomarkers to improve biological interpretability and discrimination.

This study has several important strengths. First, the final model combines AI-derived quantitative imaging features with key clinical variables. Although systemic inflammatory markers were systematically assessed as potential predictors, they did not retain independent significance after multivariable adjustment, resulting in a biologically informed yet streamlined model. Second, the use of a fully automated feature quantification platform (InferRead CT Lung) improves objectivity, enhances reproducibility, and reduces operator-dependent bias. Third, we provide an open-access, web-based nomogram to support immediate clinical implementation. Finally, the model underwent optimism-corrected bootstrap validation (1,000 resamples) and DCA, demonstrating strong predictive performance and greater standardized net benefit than treat-all or treat-none strategies across clinically relevant thresholds (0.10–0.45). Complete logistic regression coefficients and odds ratios are reported to ensure transparency.

This study also has some limitations. The single-center, retrospective design may introduce selection bias and limit generalizability, highlighting the need for external validation in prospective, multicenter cohorts (43, 44). Notably, despite the excellent calibration performance observed (slope = 0.98, intercept = 0.02), a key methodological limitation remains that predictor selection was performed once in the full cohort prior to bootstrap validation, rather than being embedded within the resampling process; this approach may still lead to a slight underestimation of model optimism. In addition, the malignancy-enriched surgical cohort may yield miscalibrated absolute risks if the model is applied to unselected nodules, underscoring the need for external validation and potential recalibration. Relatedly, the relatively high malignancy prevalence even in the lower predicted-risk stratum in our cohort indicates that the model’s absolute risks and “low-risk” interpretation may not generalize to low-prevalence populations without recalibration (36, 37). We were unable to uniformly capture CT-defined nodule location (central vs. peripheral) across the study period, which may introduce residual confounding related to histology and patient demographics. Because the model was developed using a Chinese patient population, its performance in other ethnic and geographic settings requires evaluation and potential recalibration (12). The model does not include molecular biomarkers; incorporating liquid biopsy or genomic data could improve predictive accuracy and biological interpretability (16). In addition, imaging predictors were limited to AI-extracted quantitative metrics rather than high-dimensional texture-based radiomics; future work could evaluate whether adding higher-dimensional radiomic or deep-learning features further improves performance. Head-to-head comparison with established models was not feasible due to unavailable predictors; we benchmarked against predefined baseline models. Moreover, comparisons with penalized or non-linear machine-learning approaches were not performed and should be assessed in future studies with external validation (45). Finally, because the model relies on a proprietary commercial AI platform, cross-software reproducibility requires further validation.

This study introduces a practical tool for preoperative risk stratification of indeterminate PNs ≤ 3 cm—an AI-assisted clinico–quantitative imaging nomogram that leverages AI for automated feature extraction within an interpretable logistic regression framework. By combining automated imaging quantification with clinical data, the model may support individualized decision-making primarily in surgically selected, malignancy-enriched cohorts. Its applicability to screening or incidental nodule populations requires further validation and recalibration. Future work should prioritize external validation, ideally in multicenter settings, and recalibration as needed in independent, unselected nodule cohorts. Integration of molecular biomarkers may further improve performance.

## Data Availability

The data analyzed in this study is subject to the following licenses/restrictions: Any researchers interested in this study could contact Yingding Ruan to request the data. Requests to access these datasets should be directed to Yingding Ruan. E-mail:ruanyingding@sina.com.
