# Supplementary material for: AI-assisted clinico–quantitative imaging nomogram for preoperative malignancy risk in solid and part-solid pulmonary nodules ≤ 3 cm: development and internal validation
Source: Front Oncol. 2026 Apr 13;16:1754582. doi: 10.3389/fonc.2026.1754582 (PMC13111385; doi:10.3389/fonc.2026.1754582)
Supplement: Supplementary file 1 [file DataSheet1.docx]

Implementation of the AI-Enhanced Clinico-Radiomic Prediction Model

This document provides the complete equation for calculating the linear predictor (LP) of the malignancy risk model, along with variable coding instructions.

**1. Linear Predictor (LP) Calculation**

The linear predictor is calculated using the following formula, based on the multivariable logistic regression coefficients:

LP = -0.1678 + (0.5920 × [Sex]) - (0.6294 × [Symptoms at Detection]) - (0.5956 × [Time to Surgery]) - (0.6120 × [Nodule Appearance]) - (1.1155 × [Consolidation-to-Tumor Ratio]) + (2.1311 × [Suspicious Radiologic Features]) + (0.0461 × [Nodule Size: >1-2cm]) + (1.1968 × [Nodule Size: >2-3cm]) - (0.1968 × [Min CT Attenuation / 100]) - (0.0489 × [Max CT Attenuation / 100])

**2. Variable Coding Scheme**

Categorical variables should be coded as 1 if the condition is "Yes" or "Present", and as 0 if "No" or "Absent" (or according to the reference category below).

| Variable | Coding Instruction | Coefficient to Multiply |
| --- | --- | --- |
| Intercept | N/A | -0.1678 |
| Sex | 1 = Female; 0 = Male | + 0.5920 |
| Symptoms at Detection | 1 = Present; 0 = Absent | - 0.6294 |
| Time to Surgery | 1 = ≥2 years; 0 = <2 years | - 0.5956 |
| Nodule Appearance | 1 = Solid; 0 = Part-solid | - 0.6120 |
| Consolidation-to-Tumor Ratio | 1 = >0.5; 0 = 0.01-0.50 | - 1.1155 |
| Suspicious Radiologic Features | 1 = Present; 0 = Absent | + 2.1311 |
| Nodule Size | >1-2cm 1 = Yes; 0 = No  (Reference: ≤1cm) | + 0.0461 |
| Nodule Size | >2-3cm 1 = Yes; 0 = No  (Reference: ≤1cm) | + 1.1968 |
| Min CT Attenuation (HU) | Enter the value, then divide by 100 | - 0.1968 |
| Max CT Attenuation (HU) | Enter the value, then divide by 100 | - 0.0489 |

**3. Calculating the Probability of Malignancy**

After calculating the Linear Predictor (LP), the final probability of malignancy is obtained using the following logistic function:

Probability of Malignancy = 1 / (1 + e^(-LP))

Where e is the base of the natural logarithm (approximately 2.71828).

**4. Implementation Note**

For clinical convenience, this model is also implemented as an interactive web calculator, available at: [https://ruanyingding.shinyapps.io/myshinyapp/](https://ruanyingding.shinyapps.io/myshinyapp/)
