# Supplementary material for: AI-assisted clinico–quantitative imaging nomogram for preoperative malignancy risk in solid and part-solid pulmonary nodules ≤ 3 cm: development and internal validation
Source: Front Oncol. 2026 Apr 13;16:1754582. doi: 10.3389/fonc.2026.1754582 (PMC13111385; doi:10.3389/fonc.2026.1754582)
Supplement: Supplementary file 2 [file Table1.docx]

| Supplementary Table S1. Multivariable logistic regression: variance inflation factors (VIF) for candidate predictors | | | |
| --- | --- | --- | --- |
| Variables | GVIF | DF | GVIF^(1/(2×DF)) |
| Sex | 1.05 | 1 | 1.03 |
| Age | 1.11 | 1 | 1.05 |
| Symptoms_at_Detection | 1.06 | 1 | 1.03 |
| Time_from_Detection_to_surgery | 1.07 | 1 | 1.03 |
| Nodule_Radiographic_Appearance | 2.29 | 1 | 1.51 |
| Consolidation_to_tumor | 2.01 | 1 | 1.42 |
| Suspicious_Radiologic_Features | 1.21 | 1 | 1.10 |
| Nodule_Size | 1.73 | 2 | 1.15 |
| Max_CT_Attenuation_100 | 1.10 | 1 | 1.05 |
| Min_CT_Attenuation_(per_100_HU) | 1.90 | 1 | 1.38 |
| Nodule_Volume_1000 | 1.88 | 1 | 1.37 |
| Nodule_Area_500 | 1.79 | 1 | 1.34 |
| Preoperative_SII_100 | 1.04 | 1 | 1.02 |
| **Notes.**   - GVIF: generalized variance inflation factor; DF: degrees of freedom. - “Adjusted GVIF” accounts for DF and is computed as GVIF^(1/(2×DF)). - Variables with a scale tag (e.g., “per 100 HU”, “scaled by 1,000”) were rescaled for model stability and interpretability. - All adjusted GVIF values are ≤1.51, suggesting no concerning multicollinearity among predictors. | | | |
