# Supplementary material for: AI-assisted clinico–quantitative imaging nomogram for preoperative malignancy risk in solid and part-solid pulmonary nodules ≤ 3 cm: development and internal validation
Source: Front Oncol. 2026 Apr 13;16:1754582. doi: 10.3389/fonc.2026.1754582 (PMC13111385; doi:10.3389/fonc.2026.1754582)
Supplement: Supplementary file 3 [file Table2.docx]

| Supplementary Table 2. Internal benchmarking of the full clinico–quantitative imaging model versus baseline models in the same cohort (n = 951) | | | |
| --- | --- | --- | --- |
| Model | Predictors Included | AUC (95% CI) | *P* value vs. Full Model |
| Full clinico–quantitative imaging model | Sex, symptoms at detection, time to surgery, nodule type, CTR, suspicious radiologic features, nodule size, min/max CT attenuation | 0.836 (0.804 – 0.869) | Reference |
| Clinical-only model | Sex, age, smoking history, symptoms at detection, time to surgery | 0.666 (0.624 – 0.707) | *P* < 0.001 |
| Imaging baseline model | Nodule type, CTR, suspicious radiologic features, nodule size, min/max CT attenuation | 0.822 (0.785 – 0.855) | *P* = 0.017 |
| Guideline-like imaging model | Nodule size, nodule type, CTR | 0.733 (0.693 – 0.770) | *P* < 0.001 |
| AUC, area under the ROC curve; CI, confidence interval; CTR, consolidation-to-tumor ratio; CT, computed tomography. P values: DeLong test vs. full model (reference). | | | |
